# Congenital myelomeningocele – do we have to change our management?

**DOI:** 10.1186/1743-8454-7-S1-S16

**Published:** 2010-12-15

**Authors:** Steffi Mayer, Margit Weißer, Holger Till, Gerd Gräfe, Christian Geyer

**Affiliations:** 1 Department of Pediatric Surgery University Hospital LeipzigLiebigstrasse 20a 04103 Leipzig, Germany

## Background

Eagerly awaiting the results of the Management of Myelomeningocele Study (MOMS) with an increasing interest in setting up intrauterine myelomeningocele repair (IUMR), the optimal management of patients suffering from congenital myelomeningocele (MMC) has become a matter of debate again. We performed a cross-sectional study at our referral-center for MMC to compare the outcomes of our expectantly managed patients with the results of IUMR and historical controls on which the MOMS trial has been based on.

## Materials and methods

A computed chart review revealed 71 patients suffering from spina bifida. Of those, 10 have been lost for follow-up and 2 were excluded because they had undergone IUMR. A retrospective analysis was performed only in patients that underwent MMC repair within the first two days of life and were seen at our outpatient clinic between 2008 and 2009 for a regular interdisciplinary follow-up. Data were collected on: gestational age (GA) and weight at birth, shunt status at the first year of live and age at shunt placement, radiological presence of Arnold-chiari II malformation (ACMII) and tethered cord (TC), bladder function, lower leg function and educational level. Data were compared to published results for IUMR and historical controls [1,2]. Data are given in percent or mean (standard deviation).

## Results

We analyzed the data of 43 patients born with mainly lumbosacral MMCs between 1979 and 2009 that are now 13.3 (8.9) years of age. At birth, mean GA was 264.5 (16.3) days and mean weight was 2921.3 (760.3) g, both significantly than in IUMR patients. 69.8 % required a shunt placement at a mean age of 16.0 (10.7) days, which was significantly better than historical controls. In 57.1 % an ACMII and in 41.9% a TC was observed radiologically. Only two patients underwent a surgical correction for TC. 69.7% of the patients perform clean intermittent catheterization. 56.4% are (assistant) walkers and 64.1% attend regular classes, both comparable to historical controls.

## Conclusions

With a close and interdisciplinary management by pediatric surgeons, neurologists and urologists, long-term outcomes of patients suffering from MMC can currently be considered satisfying. With respect to the known drawbacks of fetal interventions for mother and child, especially preterm delivery, the results of the MOMS trial should be awaited with caution before jumping on a complex intervention like IUMR.
